# Differences in Investigator-Initiated Trials between Japan and Other Countries: Analyses of Clinical Trials Sponsored by Academia and Government in the ClinicalTrials.gov Registry and in the Three Japanese Registries

**DOI:** 10.1371/journal.pone.0148455

**Published:** 2016-02-05

**Authors:** Tatsuya Ito

**Affiliations:** Department of Experimental Therapeutics, Institute for Advancement of Clinical and Translational Science, Kyoto University Hospital, Kyoto, Japan; National Cerebral and Cardiovascular Center, JAPAN

## Abstract

**Background:**

Following the amendment of the Pharmaceutical Affairs Law in Japan in 2003 researchers were permitted to begin investigator-initiated trials (IITs). In subsequent years, however, the number of IITs remained low. In other countries in Asia as well as in Europe, North America, and South Africa, the number of IITs has increased over the past decade. The differences in the characteristics of IITs between Japan and other countries are unknown. Some studies have analyzed the characteristics of all clinical trials according to registry databases, but there has been less research focusing on IITs.

**Aims:**

The purpose of this study is to analyze the characteristics of IITs in the ClinicalTrials.gov registry and in the three Japanese registries, to identify differences in IITs between Japan and other countries.

**Methods:**

Using Thomson Reuters Pharma™, trials sponsored by academia and government as IITs in 2010 and registered in ClinicalTrials.gov were identified. IITs from 2004 to 2012 in Japan were identified in the three Japanese registries: the University Hospital Medical Information Network Clinical Trials Registry, the Japan Pharmaceutical Information Center Clinical Trials Information, and the Japan Medical Association Center for Clinical Trials, Clinical Trials Registry. Characterization was made of the trial purposes, phases, participants, masking, arms, design, controls, and other data.

**Results:**

New and revised IITs registered in ClinicalTrials.gov during 2010 averaged about 40% of all sponsor-identified trials. IITs were nearly all early-phase studies with small numbers of participants. A total of 56 Japanese IITs were found over a period of 8 years, and these were also almost nearly all early-phase studies with small numbers of participants.

**Conclusion:**

There appear to be no great differences between Japan and other countries in terms of characteristics of IITs. These results should prompt a new review of the IIT environment in Japan.

## Introduction

Until 2003, investigators in Japan could not conduct clinical trials individually, whether to investigate a new medicine or a new application of approved drugs or even to research theories or promising treatments. Companies carried out such studies and then submitted an investigational new drug (IND) application to the Ministry of Health, Labour, and Welfare (MHLW). Amendment of the Pharmaceutical Affairs Law in July 2002 permitted investigators to apply for INDs in July 2003, thus marking the start of investigator-initiated trials (IITs) in Japan. However, the number of IIT applications for INDs remained quite small at about 60 from 2004 to 2010 [1]. About half of these were trials for new applications of an approved drug, using funding schemes for the Clinical Trial Grant Program of the Japan Medical Association Center for Clinical Trials (JMACCT) [2]. The other half was trials proposed by the author, to investigate a new drug or device in an early-phase study with a small number participants and using government funding schemes or institutional budgets. The reasons for the small number of IITs during this period include few funding opportunities and a poor quality assurance structure [3]. A review of ClinicalTrials.gov, one of the largest open registries of publicly and privately supported clinical trials worldwide, showed that there were over 7,000, registered studies from 2000 to 2010 sponsored by the United States National Institutes of Health (NIH) and US government agencies and excluding industry, which includes the majority of IITs [4]. These results highlight a large difference between Japan and other countries in terms of numbers of IITs. Key factors that may contribute to increasing IITs in Japan might be found by investigating IITs registered in ClinicalTrials.gov. Califf et al. found that trials registered in ClinicalTrials.gov exhibited heterogeneity in their methodological approaches, and that sponsored-identified trials accounted for a total of 40% of registered trials from 2007 to 2010 [4]. However, no studies have focused on IITs sponsored by academic or government institutions. Thus, the purpose of this study is to identify differences between Japan and other countries through analyzing the characteristics of trials sponsored by academia or government in the ClinicalTrials.gov database and sponsored-identified IITs in the three Japanese registries database.

The ClinicalTrials.gov registry was established in 2000 by the National Library of Medicine on behalf of the NIH. ClinicalTrials.gov is a publicly available clinical trial registry and results database. Data are self-reported by sponsors via a web-based system. Any sponsor, principal investigator, or other person or organization with primary responsibility for a trial can register trials in the database. The registry includes mandatory and optional data elements such as the trial purpose, phase, enrolled participants, age group, participant sex, masking, trial assignment, number of arms, treatment allocation, and trial control groups. ClinicalTrials.gov is a not only an integrated database for clinical trials but also a useful tool to visualize trends in current trials. In 2004, the International Committee of Medical Journal Editors (ICMJE) initiated a policy requiring registration of clinical trials as a prerequisite for publication [5]. In the United States, the Food and Drug Administration Amendment Acts of 2007 requires sponsors to register clinical trials in a database [6]. This amendment resulted in a sharp increase in the number of trials registered in ClinicalTrials.gov.

In Japan, the University Hospital Medical Information Network Clinical Trials Registry (UMIN-CTR) was begun in June 2005, as the first Japanese registry accepted by the ICMJE. The second registry site, the Japan Pharmaceutical Information Center Clinical Trials Information (JapicCTI), was established in July 2005 and the third, the Japan Medical Association Center for Clinical Trials, Clinical Trials Registry (JMACCT CTR), in December 2005 [7]. The three registries are publicly available. Data are self-reported by sponsors. All three Japanese registries include elements included in ClinicalTrials.gov, such as trial purpose, study type, study design, phase, enrolled participants, age group, participant sex, trial masking, trial assignment, number of arms, treatment allocation, and trial control groups. Under its funding rules of 2009, sponsors must register clinical trials funded by the MHLW in a trial database.

The methods used in this study are described in Section 2 (Materials and Methods). Section 3 (Results) elaborates all analyzed trials for 2010 and characterization according to three therapeutic areas. Discussion is presented in Section 4 (Discussion) and conclusions in Section 5 (Conclusion).

## Materials and Methods

Data of new or revised trials registered with ClinicalTrials.gov in 2010 were collected through Thomson Reuters Pharma™, a data source for pharmaceutical information owned by Thomson Reuters, which enables clinical trial data to be downloaded as a whole. Data of new IITs registered from 2004 to 2012 in Japan were collected via the UMIN-CTR, JapicCTI, and JMACCT CTR registry sites.

### Data set

Over 100,000 clinical trials were registered in ClinicalTrials.gov during 2010. Here, a total 88,819 trials were chosen via Thomson Reuters Pharma, by focusing on the main therapeutic areas including cardiovascular, endocrine, gastrointestinal, immunological, infectious, inflammatory, neoplasm, neurological, and respiratory diseases. This data set was downloaded from Thomson Reuters Pharma, between 25 and 28 March 2011, to facilitate aggregate analysis. Data were included in two XML data files comprising 88,819 clinical trials in total and 1,943 sponsor-identified trials, grouped according to three therapeutic areas: cardiovascular diseases, brain diseases within neurological diseases, and endocrine tumors within neoplasms. The reason for choosing these three disease areas is that it was of interest to the author to determine trends of trials in these treatment areas, having worked as a clinical trial manager with clinicians in these specialties. A total 38,003 new or revised trials, registered from 1 January to 31 December 2010, were extracted from the total 88,819 trials. Extracted trials were further categorized into sponsor-identified and non-sponsor-identified trials.

In line with the organization authority file of Thomson Reuters Pharma, sponsors were defined by organization type: academic institution, government, company, collective investment, government department or agency, market participant, non-government organization, supranational, or unknown. For instance, if the lead sponsoring organization was a university or research institute, the organization type was categorized as an academic institution. If the lead sponsor was a private company, small- to medium-sized enterprise, or start-up company, this was categorized as a company. Nearly 70% of all trials had a currently identified sponsoring organization in accordance with the organization authority file. Sponsor-identified trials were then divided into two categories, academia and government (A&G) or industry; all A&G sponsor-identified trials (A&G trials) here were IITs. If trials included both an academic institution and industry outfit as a sponsor and a collaborator, they were defined as sponsored by both. Data of A&G trials that had been classified as neurological diseases and neoplasms were regrouped into brain diseases and endocrine tumors, respectively. Within these therapeutic specialty data sets, a few data elements were missing because of limitations in the data set or logistical problems in obtaining analyzable information. For this regrouping, an index by Thomson Reuters Pharma was used to create specialty data sets.

A data set of IITs conducted under Good Clinical Practice (GCP) guidelines in Japan was collected using information in the UMIN, JAPIC or JMACCT registries. An IIT was defined as an IND application trial conducted by investigators, based on governmental funding program information, clinical trial information of the MHLW and JMACCT, or clinical trial information on websites of universities, hospitals, and research institutes. The IITs were also found on either the UMIN-CTR, JapicCTI, or JMACCT CTR sites. Because the number of trials in only 2010 was too small to compare with trials in ClinicalTrials.gov in the same year, all IITs in Japan during an 8-year period between January 2004 and March 2012 were analyzed. These data were collected from 1 April 2011 to 31 March 2012 and comprised a total of 56 trials.

### Analytical methods

From the ClinicalTrials.gov database, the three main therapeutic areas of clinical trial data (brain diseases within neurological diseases, cardiovascular diseases, and endocrine tumors within neoplasms) were characterized and analyzed, using a model containing 11 characteristics: study type (interventional or observational); purpose (treatment, basic science, diagnostic, health services, natural history, prevention, screening, supportive care, training, and other); phase (0, 1, 1/2, 1b, 2, 2/3, 2a, 2b, 3, 3b, 4); number of enrolled participants (<5, 5–10, 10–20, 20–50, 50–100, 100–200, 200–500, and >500); trial age group (children, adults, older adults, or all ages); trial masking (open-label, single-blind, or double-blind); trial assignment (single, parallel, crossover, or factorial); number of arms (1, 2, 3, 4, 5, or 6); trial allocation (randomized or non-randomized); trial control (placebo, active, dose comparison, historical, or uncontrolled); and sex of the study population (male, female, or both).

All Japanese IIT from 2004 to 2012 were characterized and analyzed, using a model containing 11 characteristics: study type (interventional or observational); purpose (treatment, prevention, pharmacokinetics, and others); phase (1, 1/2, 2, 2/3, 3 or 4); number of enrolled participants (<5, 5–10, 10–20, 20–50, 50–100, 100–200, 200–500 or >500); trial age group (children, adults, older adults, or all ages); trial masking (open-label, single-blind, or double-blind); trial assignment (single, parallel or factorial); number of arms (1, 2, 3, 4 or 5); trial allocation (randomized or non-randomized); trial control (placebo, active, dose comparison, historical or uncontrolled); and sex (male, female, or both).

Cross analyses were then performed between trial purpose and phase, between trial purpose and participants, and between trial phase and participants using ClinicalTrials.gov data and Japanese registry data, respectively.

All assessments are described as a number or a percentage. Statistical analysis software was not used in this study.

## Results

A total 38,003 of 88,819 new or revised clinical trials registered in ClinicalTrials.gov in 2010 were extracted (Table 1). Of these, 29,200 (76.8%) were identified as trials sponsored by an organization. There were 11,737 (40.2%) A&G trials. By contrast, industry-sponsored trials accounted for 19,638 (67.3%). Between 5.5% and 9.2% of trials for each therapeutic group were A&G and industry-sponsored trials. When examining all trials registered in ClinicalTrials.gov in 2010, neoplasm trials had the highest ratios, accounting for 4,705 of 8,132 (57.9%) of A&G trials. The highest number of industry-sponsored trials was for inflammatory disease, with 1,847/2,169 (85.2%).

**Table 1:** Characteristics of Studies Registered in ClinicalTrials.gov During 2010.

	All Studies	Newly Registered or Revised Studies	With Sponsor	With Sponsor (%)	Academic and Government Sponsored Studies	Academic and Government Sponsored Studies (%)	Industry-Sponsored Studies	Industry Sponsored Studies (%)	Both (%)
**Cardiovascular Disease**	5779	2492	1781	71.5%	599	33.6%	1321	74.2%	7.8%
**Endocrine Disease**	8166	3542	2819	79.6%	1072	38.0%	1933	68.6%	6.6%
**Gastrointestinal Disease**	11451	4906	3772	76.9%	1372	36.4%	2660	70.5%	6.9%
**Immunological Disease**	8879	3615	2870	79.4%	1110	38.7%	1968	68.6%	7.2%
**Infection**	9240	3775	2608	69.1%	793	30.4%	2022	77.5%	7.9%
**Inflammatory Disease**	7156	2857	2169	75.9%	450	20.7%	1847	85.2%	5.9%
**Neoplasm**	22940	10077	8132	80.7%	4705	57.9%	4178	51.4%	9.2%
**Neurological Disease**	7973	3537	2667	75.4%	907	34.0%	1908	71.5%	5.5%
**Respiratory Disease**	7235	3202	2382	74.4%	729	30.6%	1801	75.6%	6.2%
**Total**	88819	38003	29200	76.8%	11737	40.2%	19638	67.3%	

The number of A&G trials in the three therapeutic areas investigated was 385, 559, and 959 for brain disease, cardiovascular disease, and endocrine tumors, respectively (Table 2). The study type was interventional for all three therapeutic specialties, and the purpose was mostly treatment. The number of prevention trials for cardiovascular disease, 78/599 (13%), was higher than for the other two areas. Phases 0 through 2 accounted for over 60% and 80% for brain diseases and endocrine tumors trials, respectively; however, these phases represented only about 35% of cardiovascular trials. The category of 20–50 enrolled participants was most frequent for the three therapeutic areas: 125/385 (32.5%), 147/599 (24.5%), and 365/959 (38.1%) for brain disease, cardiovascular disease, and endocrine tumors, respectively. Interestingly, cardiovascular disease trials had the largest sized trials, with 200–500 (83/599, 13.9%) and >500 (75/599, 12.5%) enrolled participants. The adult age group was the most frequent of all groups, but there were 67/385 (17.4%) brain disease trials conducted in children. Masking was completely different among the three trial areas. Open-label was the most frequent for brain disease and endocrine tumor trials, with 165/385 (42.9%) and 598/959 (62.4%), respectively. However, double-blind was most prevalent in cardiovascular disease trials, with 275/599 (45.9%). Assignment also differed among the three therapeutic areas. In brain disease trials, single and parallel groups accounted for 88/385 (22.9%) and 93/385 (24.2%), respectively, however, missing data represented about half of brain disease trials, or 187/385 (48.6%). In cardiovascular disease trials, parallel groups accounted for 314/599 (52.4%). In endocrine tumor trials, single groups accounted for 282/959 (29.4%), but missing data accounted for 587/959 (61.2%) trials. Trial arms were predominantly (with about 60%) one- and two-armed trials among the three therapeutic areas. One-armed trials were the most frequent among brain disease and endocrine tumor trials, with 116/385 (30.1%) and 409/959 (42.6%), respectively. In cardiovascular disease trials, two-armed trials accounted for 275/599 (45.9%). Allocation differed by specialty. Cardiovascular disease trials were mostly randomized, with 472/599 (78.8%), whereas brain disease and endocrine tumor trials were less so, with 142/385 (36.9%) and 255/959 (26.6%), respectively. However, there were missing data for about 50% of trials for the latter two specialties. The trial control was also different among these areas. Brain disease and cardiovascular disease trials were mostly conducted using placebo controls, with 83/385 (21.6%) and 245/599 (40.9%), respectively. Endocrine tumor trials were mostly performed using active controls, with 133/959 (13.9%). However, over 50% of brain disease and endocrine tumor trials had missing data. Trials included both sexes, except for endocrine tumor trials; half of these were for breast or ovarian cancers and therefore included only females.

**Table 2:** Characteristics of A&G Studies Registered in ClinicalTrials.gov During 2010.

		Brain Disease within Neurological Disease (%) (n = 385)	Cardiovascular Disease (%) (n = 599)	Endocrine Tumor within Neoplasm (%) (n = 959)
**Study Type**	**Interventional**	374 (97.1)	580 (96.8)	948 (98.9)
	**Observational**	11 (2.9)	18 (3.0)	10 (1.0)
	**Missing**	0 (0.0)	1 (0.2)	1 (0.1)
**Purpose**	**Treatment**	337 (87.5)	460 (76.8)	890 (92.8)
	**Basic Science**	3 (0.8)	12 (2.0)	3 (0.3)
	**Diagnostic**	7 (1.8)	17 (2.8)	12 (1.3)
	**Health Services**	1 (0.3)	0 (0.0)	0 (0.0)
	**Natural History**	1 (0.3)	0 (0.0)	0 (0.0)
	**Other**	0 (0.0)	4 (0.7)	1 (0.1)
	**Prevention**	18 (4.7)	78 (13.0)	22 (2.3)
	**Screening**	3 (0.8)	3 (0.5)	2 (0.2)
	**Supportive Care**	8 (2.1)	8 (1.3)	18 (1.9)
	**Training**	0 (0.0)	0 (0.0)	1 (0.1)
	**Missing**	7 (1.8)	17 (2.8)	10 (1.0)
**Phase**	**Phase 0**	1 (0.3)	5 (0.8)	4 (0.4)
	**Phase 1**	75 (19.5)	32 (5.3)	192 (20.0)
	**Phase 1/2**	42 (10.9)	32 (5.3)	97 (10.1)
	**Phase 2**	116 (30.1)	87 (14.5)	479 (49.9)
	**Phase 2/3**	10 (2.6)	24 (4.0)	7 (0.7)
	**Phase 3**	33 (8.6)	82 (13.7)	122 (12.7)
	**Phase 4**	34 (8.8)	134 (22.4)	6 (0.6)
	**Phase Not Specified**	74 (19.2)	200 (33.4)	52 (5.4)
	**Missing**	0 (0.0)	3 (0.5)	0 (0.0)
**Number of Enrolled Participants**	**<5**	5 (1.3)	8 (1.3)	13 (1.4)
	**5–10**	10 (2.6)	9 (1.5)	15 (1.6)
	**10–20**	55 (14.3)	47 (7.8)	66 (6.9)
	**20–50**	125 (32.5)	147 (24.5)	365 (38.1)
	**50–100**	85 (22.1)	125 (20.9)	242 (25.2)
	**100–200**	38 (9.9)	89 (14.9)	84 (8.8)
	**200–500**	29 (7.5)	83 (13.9)	49 (5.1)
	**>500**	15 (3.9)	75 (12.5)	81 (8.4)
	**Missing**	23 (6.0)	16 (2.7)	44 (4.6)
**Trial Age Group**	**Children (0–17 y)**	67 (17.4)	37 (6.2)	11 (1.1)
	**Adults (18–65 y)**	262 (68.1)	468 (78.1)	820 (85.5)
	**Older adults (66+ y)**	9 (2.3)	3 (0.5)	5 (0.5)
	**All ages**	7 (1.8)	0 (0.0)	0 (0.0)
	**Missing**	40 (10.4)	91 (15.2)	123 (12.8)
**Trial Masking**	**Open-label**	165 (42.9)	212 (35.4)	598 (62.4)
	**Single-blind**	11 (2.9)	47 (7.8)	5 (0.5)
	**Double-blind**	99 (25.7)	275 (45.9)	49 (5.1)
	**Missing**	110 (28.6)	65 (10.9)	307 (32.0)
**Trial Assignment**	**Single Group**	88 (22.9)	95 (15.9)	282 (29.4)
	**Parallel**	93 (24.2)	314 (52.4)	82 (8.6)
	**Crossover**	15 (3.9)	58 (9.7)	8 (0.8)
	**Factorial**	2 (0.5)	14 (2.3)	0 (0.0)
	**Missing**	187 (48.6)	118 (19.7)	587 (61.2)
**Number of Arms**	**1**	116 (30.1)	92 (15.4)	409 (42.6)
	**2**	96 (24.9)	275 (45.9)	145 (15.1)
	**3**	14 (3.6)	52 (8.7)	35 (3.6)
	**4**	7 (1.8)	21 (3.5)	11 (1.1)
	**5**	1 (0.3)	1 (0.2)	3 (0.3)
	**6**	1 (0.3)	4 (0.7)	2 (0.2)
	**Missing**	150 (39.0)	154 (25.7)	354 (36.9)
**Trial Allocation**	**Randomized**	142 (36.9)	472 (78.8)	255 (26.6)
	**Non-randomized**	52 (13.5)	52 (8.7)	218 (22.7)
	**Missing**	191 (49.6)	75 (12.5)	486 (50.7)
**Trial Control**	**Placebo Control**	83 (21.6)	245 (40.9)	44 (4.6)
	**Active Control**	39 (10.1)	131 (21.9)	133 (13.9)
	**Dose Comparison**	13 (3.4)	25 (4.2)	19 (2.0)
	**Historical Control**	4 (1.0)	6 (1.0)	21 (2.2)
	**Uncontrolled**	24 (6.2)	41 (6.8)	73 (7.6)
	**Missing**	222 (57.7)	151 (25.2)	669 (69.8)
**Sex**	**Female Only**	8 (2.1)	34 (5.7)	468 (48.8)
	**Male Only**	4 (1.0)	22 (3.7)	7 (0.7)
	**Both**	373 (96.9)	543 (90.7)	484 (50.5)

**Fig 1:**
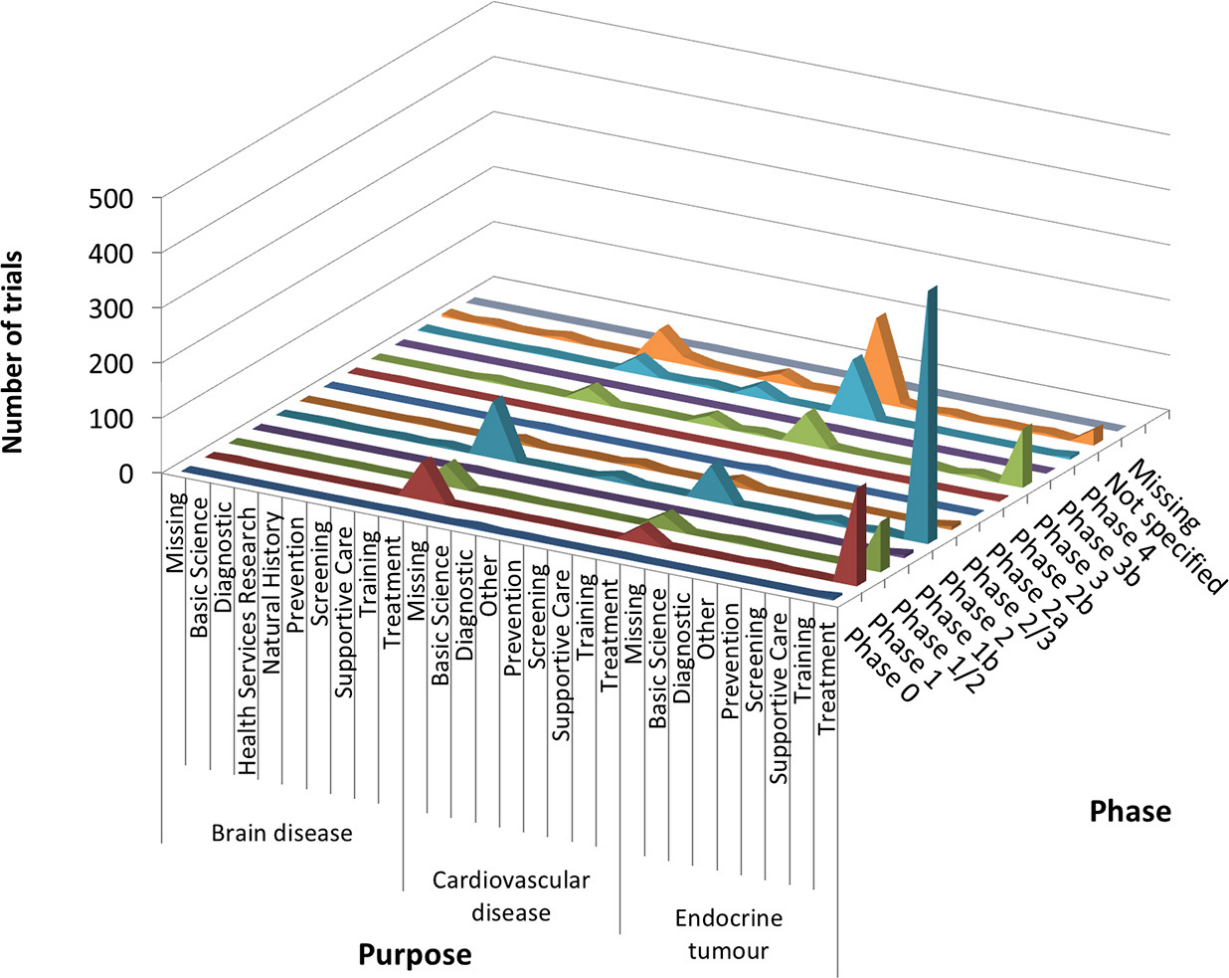
Cross-analyses between trial purpose and phase in ClinicalTrials.gov. Curve areas represent the number of trials across the phase and the trial purposes, for brain diseases, cardiovascular disease, and endocrine tumors, respectively.

**Fig 2:**
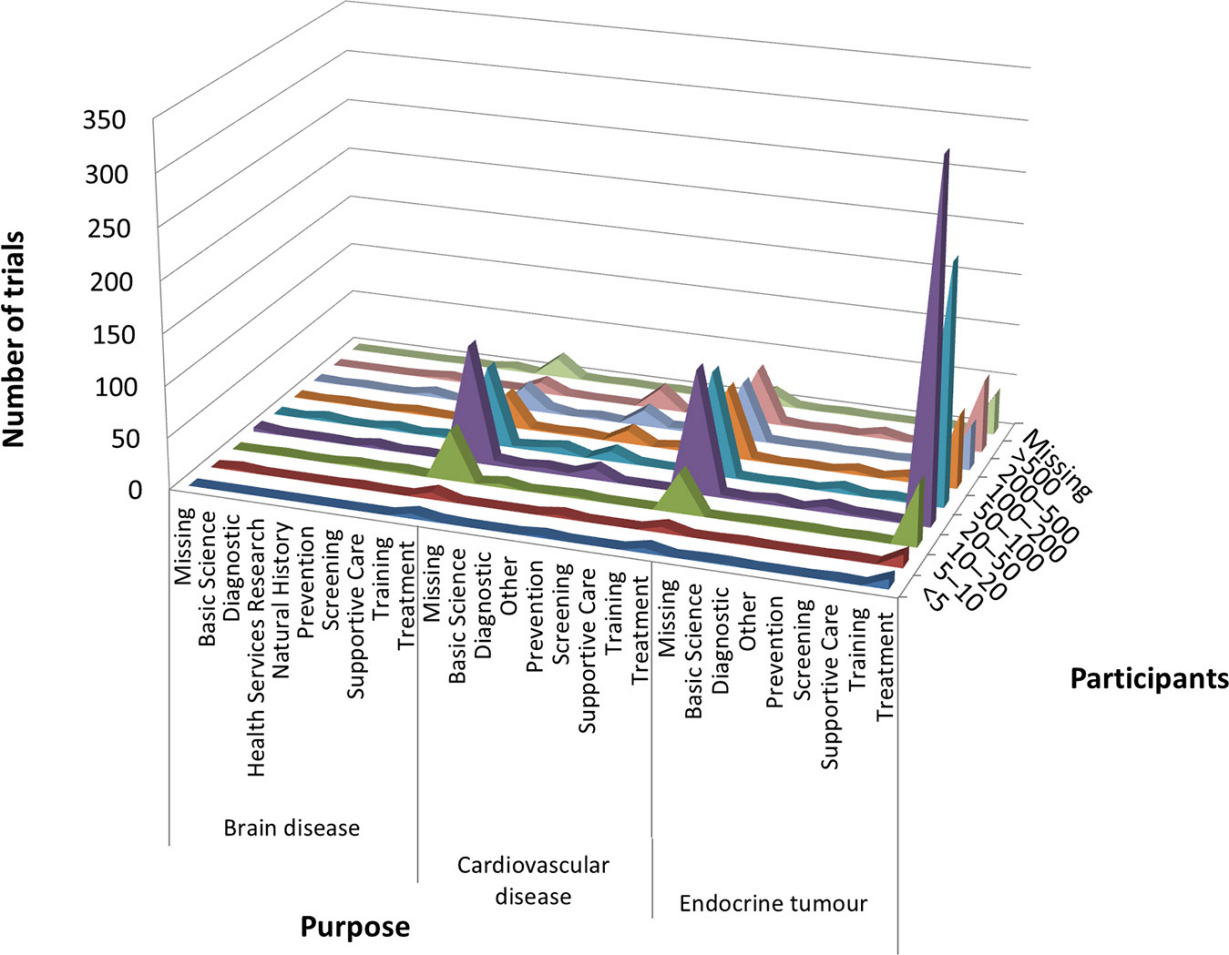
Cross-analyses between trial purpose and participants in ClinicalTrials.gov. Curve areas represent the number of trials across the participants and the trial purposes, for brain diseases, cardiovascular disease, and endocrine tumor, respectively.

**Fig 3:**
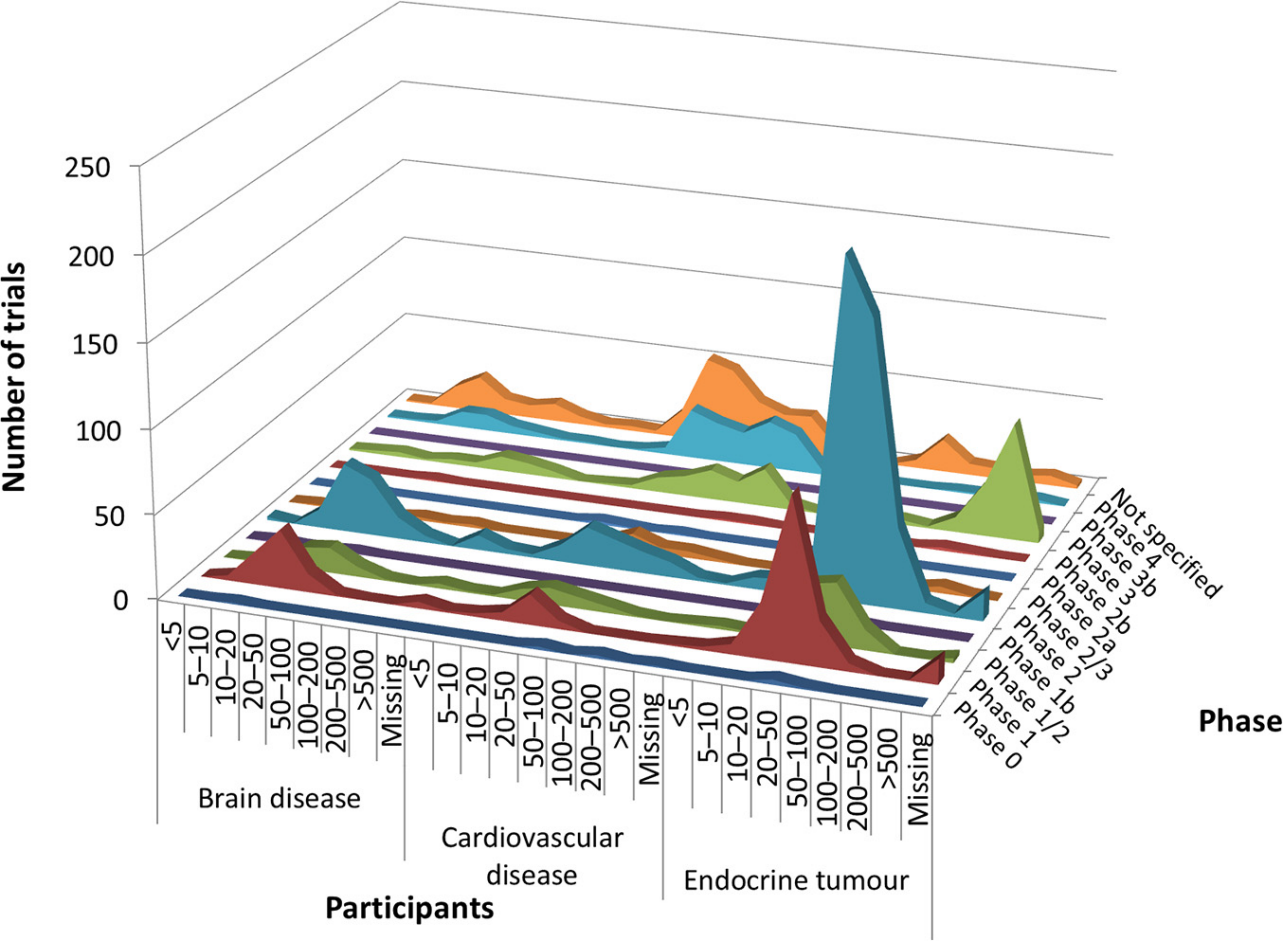
Cross-analyses between trial participants and phase in ClinicalTrials.gov. Curve areas represent the number of trials across the phase and the participants, for brain diseases, cardiovascular disease, and endocrine tumor, respectively.

According to cross-analysis between trial purposes and phases, most brain disease and endocrine tumor trials were for treatment and from early phase through phase 2 (Fig 1). Cardiovascular disease trials for the purpose of treatment showed two peaks, one for early phase (phases 1 to 2) and another for late phase (phases 3 to 4) trials. In the cross-analysis between trial purpose and participants, all three therapeutic areas were dominated by low enrollment numbers (20–50 participants) and were for the purpose of treatment (Fig 2). Cross-analysis between trial phases and participants identified that A&G trials were nearly all early phase (through phase 2) with fewer than 100 enrolled participants (Fig 3). However, in all three specialties, there were also some peaks in late-phase trials with large enrollment.

**Fig 4:**
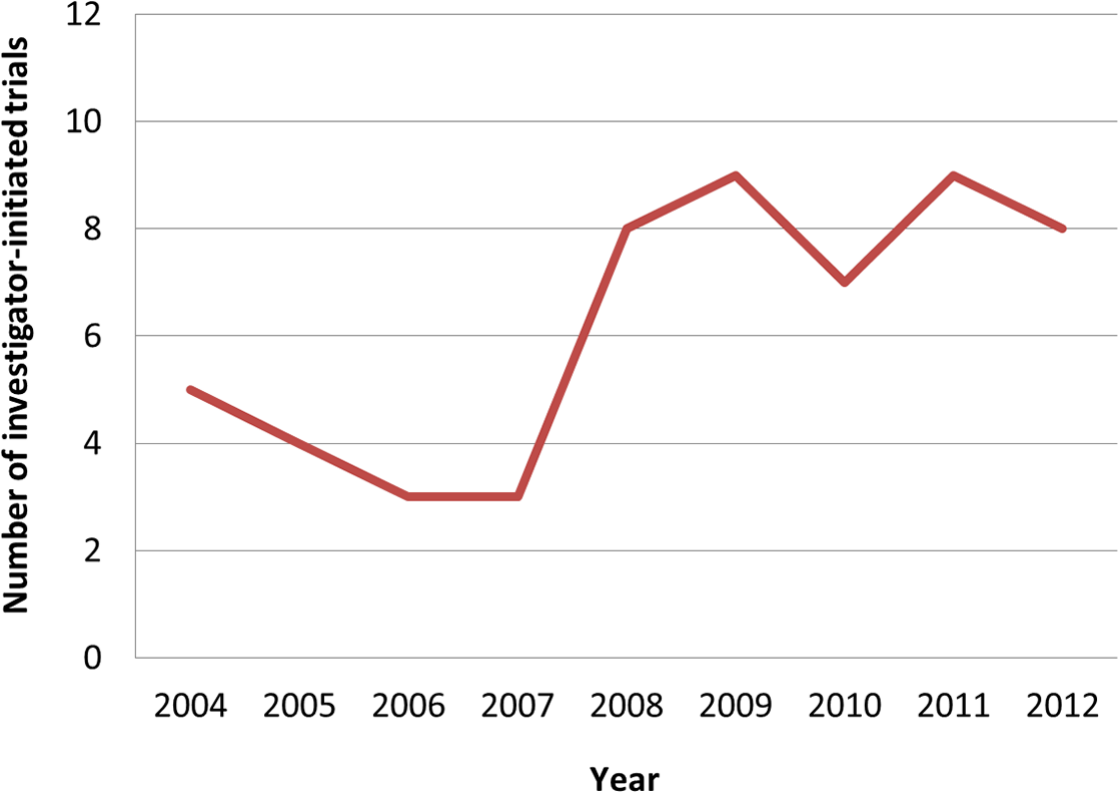
Number of investigator-initiated trials in Japanese registries. Curve represents the number of investigator-initiated trials from 2004 to 2012.

In Japan, IITs under GCP began in 2004. A total of 56 IITs were found over the 8 years from January 2004 to March 2012 in the three Japanese registries (Fig 4). There were 29, 24, and three IITs identified in the UMIN-CTR, JMACCT CTR, and JapicCTI registries, respectively. The number of IITs subsequently showed a slight annual increase, despite decreasing for 2 years between 2006 and 2007. Table 3 shows the characteristics of the 56 identified IITs, analyzed according to the 11 categories. The study type was dominated by interventional for all trials, and the purpose was mostly treatment. The number of prevention trials and pharmacokinetics trials were as follows: four influenza vaccine studies, and three phase 1 and 1/2 studies during the study period, respectively. Phases 1 to 2 accounted for around 50% of all trials, and phase 3 trials represented 12/56 (21.4%). Studies in which the phase was not specified were the mostly exploratory trials of medical devices. The category of 20–50 enrolled participants was the most frequent, with 26/56 (46.4%). Trial age groups varied widely, but children, adults and older adults were the groups most frequently represented, together accounting for 21/56 (37.5%). Open-label was the most frequent type of trials masking, with 42/56 (75.0%). Assignment was predominantly single with 33/56 (58.9%) and parallel with 21/56 (35.7%). Trial arms were mostly one-armed trials with 32/56 (57.1%) and two-armed trials with 19/56 (33.9%). Allocation was dominated by non-randomized trials with 35/56 (62.5%), followed by randomized trials with 20/56 (35.7%). In terms of the trial control, uncontrolled studies were the most prevalent with 33/56 (58.9%), followed by placebo-control with 7/56 (12.5%), active control with 6/56 (10.7%), dose comparison with 7/56 (10.7%), and historical control with 4/56 (7.1%). Most trials included both sexes nearly equally, except for female-only breast or ovarian cancer trials and male-only trials for male hormonal disorders and pharmacokinetics.

**Table 3:** Characteristics of Investigator Initiated Trials Registered in Japan from 2004 to 2012.

		Number of IIT (%) (n = 56)
**Study Type**	**Interventional**	55 (98.2)
	**Observational**	1 (1.8)
**Purpose**	**Treatment**	48 (85.7)
	**Prevention**	4 (7.1)
	**Pharmacokinetic**	3 (5.4)
	**Other**	1 (1.8)
**Phase**	**Phase 1**	4 (7.1)
	**Phase 1/2**	5 (8.9)
	**Phase 2**	19 (33.9)
	**Phase 2/3**	9 (16.1)
	**Phase 3**	12 (21.4)
	**Phase 4**	0 (0.0)
	**Not specified**	7 (12.5)
**Number of Enrolled Participants**	**<5**	1 (1.8)
	**5–10**	1 (1.8)
	**10–20**	9 (16.1)
	**20–50**	26 (46.4)
	**50–100**	5 (8.9)
	**100–200**	8 (14.3)
	**200–500**	6 (10.7)
	**>500**	0 (0.0)
**Trial Age Group**	**Children (0–17)**	7 (12.5)
	**Children and Adults (0–65)**	1 (1.8)
	**Children, Adults and Older adults (0–75)**	21 (37.5)
	**Adults (20–65)**	9 (16.1)
	**Adults and Older adults (20–84)**	15 (26.8)
	**Older adults (66+y)**	0 (0.0)
	**All ages**	2 (3.6)
	**Missing**	1 (1.8)
**Trial Masking**	**Open-label**	42 (75.0)
	**Single-blind**	4 (7.1)
	**Double-blind**	10 (17.9)
**Trial Assignment**	**Single Group**	33 (58.9)
	**Parallel**	21 (37.5)
	**Factorial**	1 (1.8)
	**Missing**	1 (1.8)
**Number of Arms**	**1**	32 (57.1)
	**2**	19 (33.9)
	**3**	2 (3.6)
	**4**	1 (1.8)
	**5**	1 (1.8)
	**Missing**	1 (1.8)
**Trial Allocation**	**Randomized**	20 (35.7)
	**Non-randomized**	35 (62.5)
	**Missing**	1 (1.8)
**Trial Control**	**Placebo Control**	7 (12.5)
	**Active Control**	6 (10.7)
	**Dose Comparison**	6 (10.7)
	**Historical Control**	4 (7.1)
	**Uncontrolled**	33 (58.9)
**Sex**	**Female Only**	7 (12.5)
	**Male Only**	3 (5.4)
	**Both**	46 (82.1)

**Fig 5:**
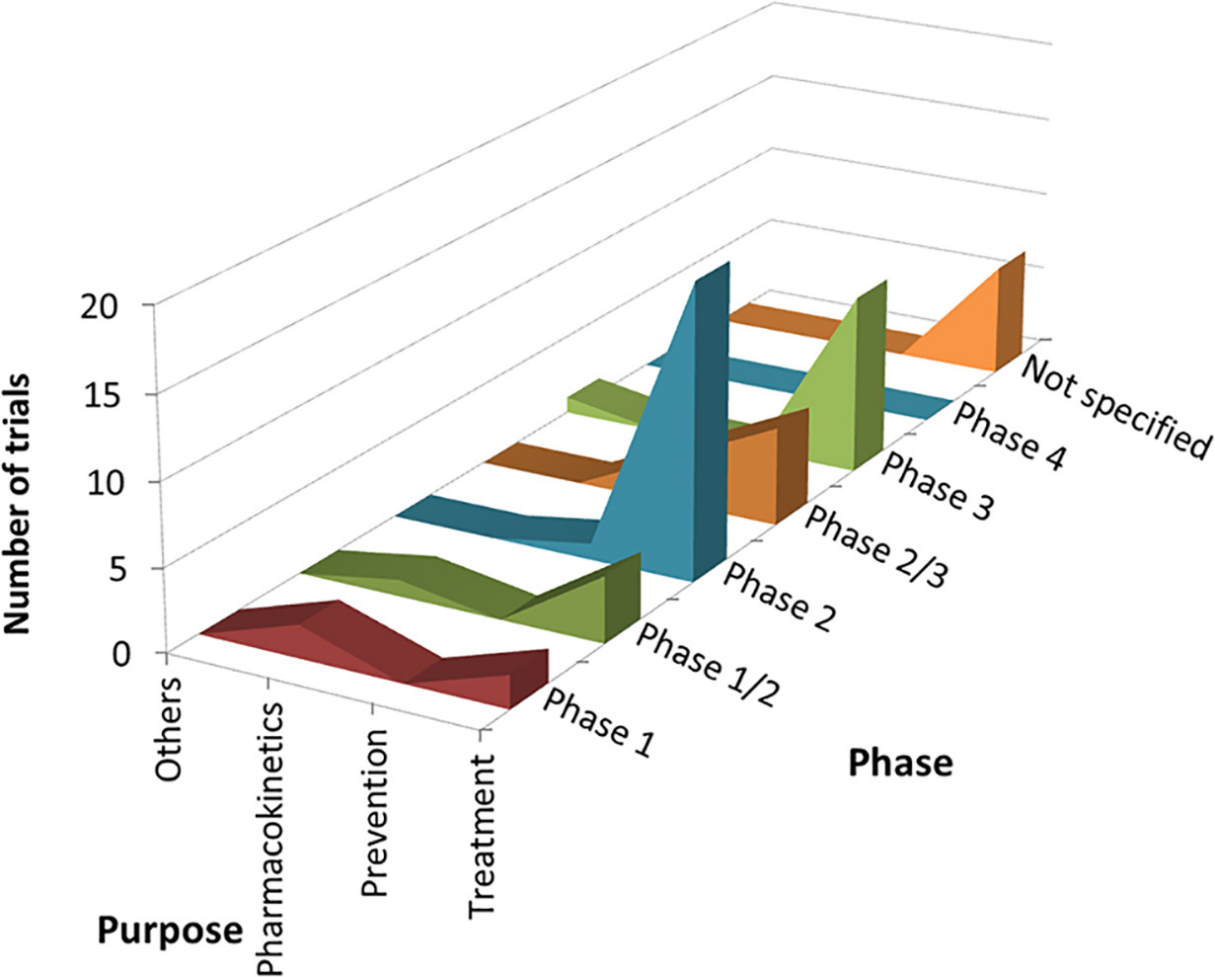
Cross-analyses between trial purpose and phase in Japanese registries. Curve areas represent the number of trials across the phase and the trial purposes.

**Fig 6:**
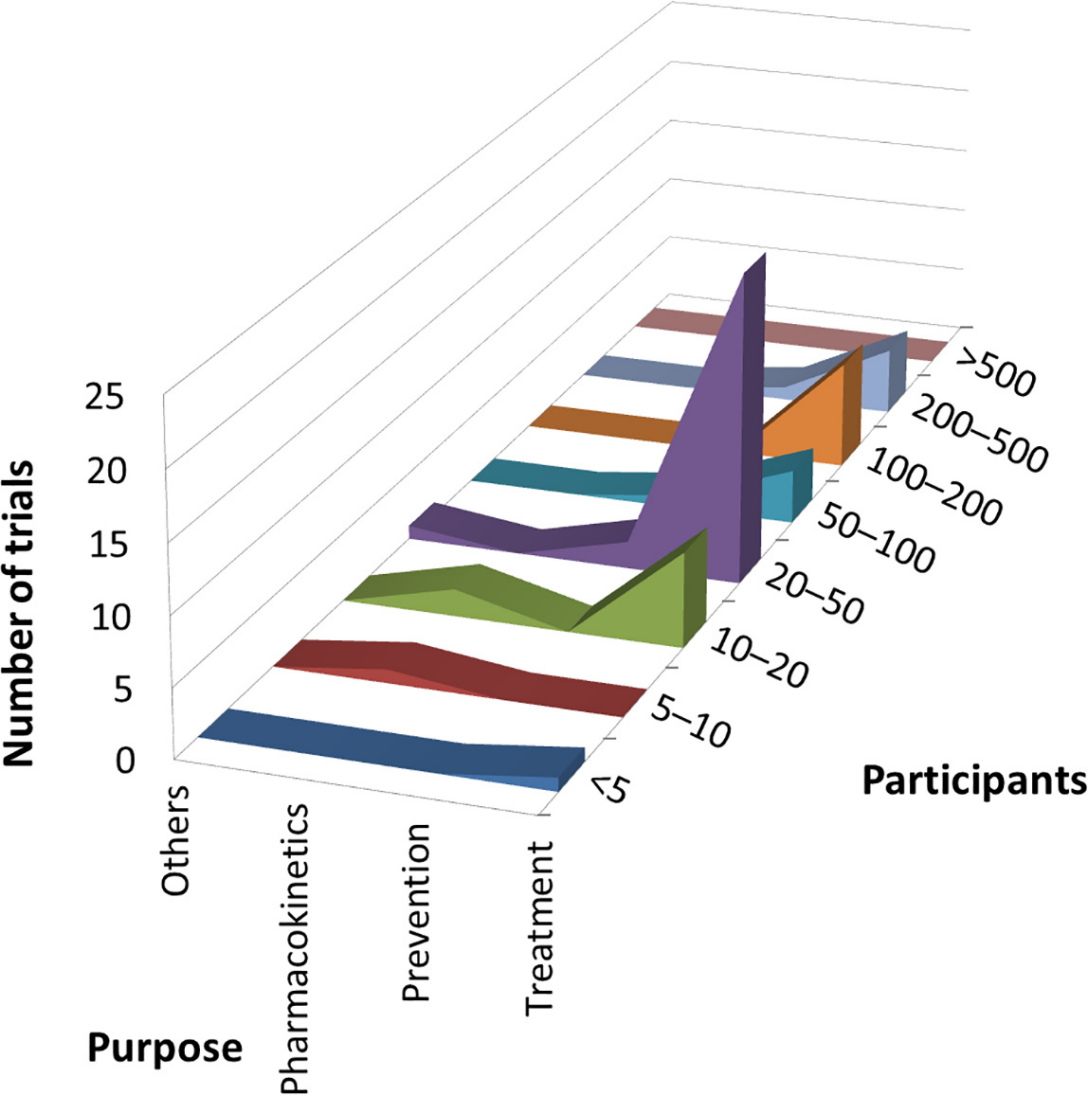
Cross-analyses between trial purpose and participants in Japanese registries. Curve areas represent the number of trials across participants and the trial purposes.

**Fig 7:**
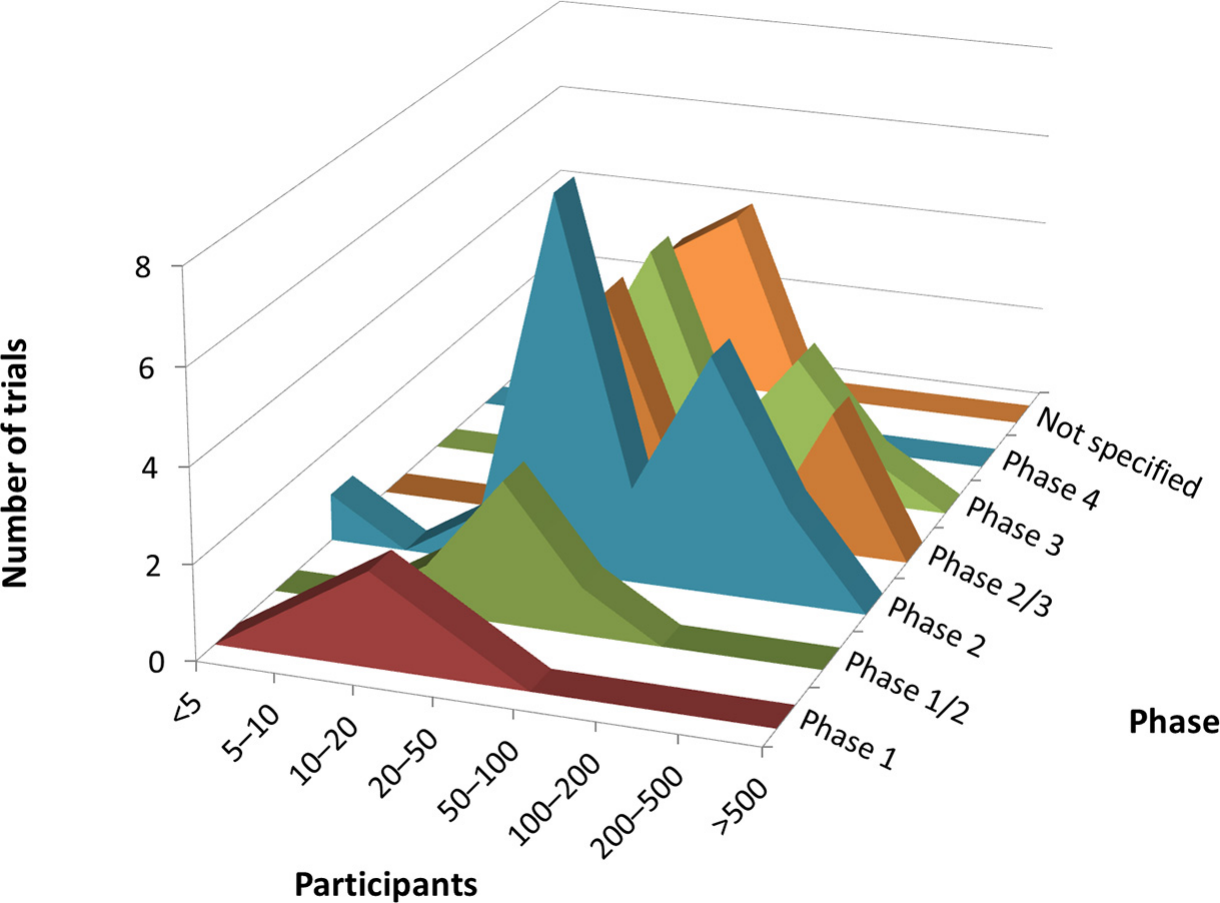
Cross-analyses between trial participants and phase in Japanese registries. Curve areas represent the number of trials across phase and the participants.

Regarding cross-analysis between trial purposes and phases, most trials were for treatment and from early phase through phase 2 (Fig 5). In the cross-analysis between trial purpose and participants, trials were dominated by those with low enrollment numbers (up to 20–50 participants) and were for the purpose of treatment (Fig 6). Cross-analysis between trial phases and participants identified that nearly all trials were early phase (through phase 2) with fewer than 100 enrolled participants (Fig 7). However, there were also some peaks in late-phase trials.

## Discussion

To identify differences between Japan and other countries regarding IITs with academic or government sponsors, this study investigated the characteristics of newly registered or revised sponsor-identified trials in ClinicalTrials.gov in 2010, as compared with characteristics of newly registered IITs in the UMIN-CTR, JapicCTI or JMACCT CTR registries from 2004 to 2012.

First, sponsor-identified trials represented over 75% of all trials registered with ClinicalTrials.gov in 2010, and A&G trials accounted for about 40% of all sponsor-identified trials. The number of A&G trials was lower than that of industry-sponsored trials. Interestingly, each therapeutic area had different percentages of trials sponsored by A&G and industry. Neoplasm trials had the most A&G trials with 57.9%, which represents efforts by academia to develop new medicines, devices, and diagnoses for this therapeutic area. Conversely, inflammatory disease trials had the least A&G trials at 20.7%, which might indicate that industry is more interested in development in this area.

According to analyses of the three therapeutic areas investigated, A&G trials included primarily small (<100 participants) and early-phase trials. This suggests that these trials are likely to be broad, exploratory ones and very heterogeneous. Brain disease and endocrine tumor areas had a concentration of early-phase trials and/or feasibility studies and were more often to be open-label, single-arm studies. Interestingly, endocrine tumor studies were particularly characterized by female-only trials for ovarian or breast cancer, which indicates an increasing trend in the development of new ovarian and breast cancer treatments in 2010. Meanwhile, the characteristics of cardiovascular disease trials revealed two trends, a tendency toward early-phase trials and/or feasibility studies, similar to the other two areas, and a trend toward observational trials with > 500 participants and the use of active controls.

In Japan, the number of IITs (56 trials over the 8-year study period) was quite a bit smaller than those registered in ClinicalTrials.gov. Japanese IITs were dominated by interventional treatment studies in early phase with few participants. About 20% of IITs were late-phase studies with more than 100 participants. Japanese IITs were mostly characterized by open-label, single group, non-randomized, and uncontrolled studies, meaning that the IITs in Japan were focused on more exploratory and feasible studies than those in the other countries. However, this conclusion is based on only about 60 trials beginning in 2004; more long-term observation studies are needed. Overall, most Japanese IITs were early phase and small trials. This characteristic appears no different to IITs in ClinicalTrials.gov. Nevertheless, there is a large difference in the number of IITs between Japan and other countries. No single factor that is likely to affect the number of Japanese IITs was found in this study. Some reasons for the low number of IITs are thought to include few funding opportunities and a poor quality assurance structure [3,8]; however it is the author’s opinion that the cause fundamentally lies within the clinical trial system in Japan. The number of Japanese IITs could be increased by improving the clinical trial environment or key areas within academia.

The first area in which Japan must improve is its clinical trial system. There are actually two IIT systems in Japan, one for IND and another for non-IND trials. IND trials are undertaken in accordance with GCP, with an IND application submitted to the MHLW. These trials are regulated and monitored in the same way as commercial trials and are reported monthly to the Institutional Review Board and the Pharmaceuticals and Medical Devices Agency in cases of safety issues. With few professionals available to operate clinical trials, such requirements present large hurdles for academia to overcome because they are often time-consuming and involve much paperwork and high costs. By contrast, non-IND trials are conducted under the Guidelines for Clinical Research issued by the MHLW in 2003, which do not require submission of an application [9]. The requirements for conducting non-IND IITs are not as stringent as those for IND trials; however, annual reporting to the research ethics committee is required, as well as to the MHLW for safety issues. Nevertheless, these requirements involve less time, less paperwork, and lower cost than IND trials. It is therefore easier for academic institutions to conduct non-IND trials, even with few staff, which has resulted in over 18,000 non-IND trials, according to the UMIN-CTR, since its establishment in 2005 [10]. The current environment is likely one reason for the small number of IITs for IND. However, non-IND trials are not performed in line with GCP. Therefore, the data from non-IND trials are not valid for new indications for approved drugs because of poorer data quality, owing to the less stringent requirements for non-IND trials regarding paperwork, research processes, and quality assurance. Conducting non-IND trials might provide some new evidence, but it is not useful in clinical practice.

Therefore, non-IND trials that do not follow GCP should be closed down immediately, and a review of the current Japanese IND-type IIT system should be carried out, with the aim to make it more efficient thereby simplifying the conduct of IND trials. Such actions could greatly improve the current clinical trial situation in Japan by increasing the number of IND-type IITs performed, through eliminating the need for clinical trial experts and the involvement of regulatory authorities. Before such implementation, however, effective ways to reduce paperwork, costs, and the time required to address regulatory issues must be considered. The Japanese government has created several national programs to build support institutions within academia that provide clinical trial services such as data management, statistical analysis, and monitoring. These programs could also be helpful for investigators in conducting IND trials [11–14].

There are some limitations in this study. One is that there were some missing data despite mandatory data requirements by ClinicalTrials.gov and the Japanese registries. Recent reports have described that the ClinicalTrials.gov database could contain some quality issues owing to self-reporting by sponsors [15]. Another limitation is the focus only on 1-year characteristics of A&G trials registered in ClinicalTrials.gov by sponsors from all over the world. In addition, there were too few Japanese IITs to clearly demonstrate trial characteristics. The EudraCT database was not open to the public at the time this article was written; therefore, trial characteristics in European countries were unavailable. A comparison that includes European registries should be made in the near future.

## Conclusion

In this study, new and revised clinical trials sponsored by A&G that were registered in ClinicalTrials.gov during 2010 averaged about 40% of all sponsor-identified trials. According to analysis of the three main therapeutic areas, these A&G trials were nearly all early phase studies for the purpose of treatment, with a small number of participants. There were around 60 Japanese IITs over an 8-year period beginning in 2004. These trials were also mostly early-phase studies for the purpose of treatment, with a small number of participants. The characteristics of IITs between Japan and other countries exhibited no great differences, although there was a large difference in the number of Japanese IITs compared with other countries. Japan’s IIT system and clinical trial environment should be immediately reviewed, to close this gap.
